# Simulation in resuscitation teaching and training, an evidence based practice review

**DOI:** 10.4103/0974-2700.70758

**Published:** 2010

**Authors:** Sandeep Sahu, Indu Lata

**Affiliations:** Department of Anaesthesiology and Maternal and Reproductive Health, Sanjay Gandhi Post Graduate Institute of Medical Sciences, Lucknow, UP, India; 1 Sanjay Gandhi Post Graduate Institute of Medical Sciences, Lucknow, UP, India

**Keywords:** Simulation, resuscitation training, cardiac arrest, education, emergency medicine, outcome

## Abstract

In the management of a patient in cardiac arrest, it is sometimes the least experienced provider giving chest compressions, intubating the patient, and running the code during the most crucial moment in that patient’s life. Traditional methods of educating residents and medical students using lectures and bedside teaching are no longer sufficient. Today’s generation of trainees grew up in a multimedia environment, learning on the electronic method of learning (online, internet) instead of reading books. It is unreasonable to expect the educational model developed 50 years ago to be able to adequately train the medical students and residents of today. One area that is difficult to teach is the diagnosis and management of the critically ill patient, specifically who require resuscitation for cardiac emergencies and cardiac arrest. Patient simulation has emerged as an educational tool that allows the learner to practice patient care, away from the bedside, in a controlled and safe environment, giving the learner the opportunity to practice the educational principles of deliberate practice and self-refection. We performed a qualitative literature review of the uses of simulators in resuscitation training with a focus on their current and potential applications in cardiac arrest and emergencies.

## INTRODUCTION

Research in adult education has identified several key characteristics of adult learners.[1] They are independent, self-directed, and internally motivated to learn; their eagerness to learn is related to the social and professional roles of their day-to-day lives; they seek immediate applications for the knowledge gained; and they have accumulated a wealth of experience that serves as a resource and foundation for their ongoing intellectual development. When training health care professionals, these expectations are best met through hands-on practice in immersive-simulated medical environments. Medical education and resident training has changed dramatically in the 21^st^century because of a dynamic shift in the attitudes of our patients and learners, as well as the creation of information technology. Patients today are no longer passive in their healthcare but are fully engaged using tools such as the Internet, to make their own care decisions. Patients also have an increased awareness to medical errors, calling into question the century-old practice of “see one, do one, teach one”.[2] Learner expectations have also changed in the current, fast-paced, technological environment: In the past, trainees would read about unfamiliar disease processes, sit passively in a lecture, and observe skilled clinicians prior to practicing on a patient. This form of learning is not conducive to adult learning or to current practice environments. Research specific to medical education has shown that adults learn faster and have greater retention of knowledge when they participate in an interactive setting.[3]

We conducted a systematic review of the literature on the use of simulators in the training of resuscitation. PUBMED was searched using the terms “simulation,” “training,” and “resuscitation.” A summary of our findings is presented with a discussion of both the advantages and disadvantages of applied simulation to training.

Patient simulation has been suggested as an ideal tool for teaching in this new generation of learners, allowing them to engage actively in their learning process while doing no harm to their patients. Review of literature shows that to get optimal outcome of resuscitation, simulation training is essentially needed not only for adult patients but also for pediatric patients.

One area of medicine needing a fundamental shift in the teaching model is in the training of cardiac emergencies. A recent study by Pebedy *et al*. of 58,593 cardiac arrest patients demonstrated a significant decline in survival rates for in-hospital cardiac arrests during nights and weekends for all areas of the hospital except the emergency department (ED) and trauma service.[4] The authors theorized that this was likely due to less direct supervision by senior staff in areas of the hospital outside of the ED and trauma center.

In the USA, the current model for training in cardiac emergencies is usually initiated at the beginning of internship with the Advanced Cardiac Life Support (ACLS) course. After taking the 2-day course, the physician becomes certified in the initial management for most cardiac emergencies such as cardiac arrest and unstable arrhythmias. Though ACLS certification has become a standard process for ensuring minimum competency for practitioners responding to cardiac emergencies, it does not require a mastery of the knowledge and skill needed to handle the complexities of advanced clinical decision-making. With evidence correlating increased patient survival to factors such as rate and quality of chest compressions and early recognition of ventricular fibrillation and defibrillation, it becomes evident that instruction through passive lectures and a one-time skills station is inadequate for providing optimal survival opportunities for these patients.[5–7] Research in instructional science has demonstrated that, in order to ensure acquisition and maintenance of a skill at the expert level, the deliberate practice of the educational objective must be employed.[8] Simulation is an ideal educational tool for the training of these high-stakes patients.

By definition simulation-based training involves immersion of a trainee in a realistic situation (scenario) created within a physical space (simulator) that replicates the real environment with fidelity sufficient to achieve suspension of disbelief on the part of the trainee. Such training has been adopted as the standard in professions characterized by highly technical, complex, dynamic environments in which crises evolve rapidly and the risk to human life is high (e.g., aerospace, the military, and nuclear power).

Gaba defines simulation as an instructional process that substitutes real patient encounters with artificial models, live actors, or virtual reality patients with the goal of replicating patient care scenarios in a realistic environment for the purposes of feedback and assessment.[9] Simulation-based methodologies were used initially in the medical field in the 1980s for training in the management of critically ill patients in the operating room.[10,11] The first high-fidelity simulation-based training program in neonatal resuscitation was developed at Stanford University in the mid-1990s.[12] Similar programs are beginning to emerge at other centers across the country and around the world. Simulation-based training has many advantages. It provides trainees with increased clinical experience because rare but devastating events can be simulated and practiced many times. It is convenient; trainees can schedule simulation sessions at times that fit into their schedules. It decreases the use of hospital resources by reducing the time spent teaching in expensive clinical environments. It is also much safer to train on patient simulators than on real patients. Many national and international organizations have recommended simulation-based resuscitation training, including the Joint Commission on Accreditation of Healthcare Organizations and the International Liaison Committee on Resuscitation.[13,14]

The development of a real-time simulator for teaching ultrasound techniques and interpretation was accomplished. Simulators have been proposed as an ideal tool for assessment of students for clinical skills. Programmed patients and simulated clinical situations, including mock disaster drills, have been used extensively for education and evaluation. These “lifelike” simulations are expensive and lack reproducibility.

## CLASSIFICATION OF SIMULATION

Currently, no standardized classification in simulation exists, but it is often divided into four areas by the educational tool: standardized patient, screen-based computer, partial-task, and high-fidelity simulator. Standardized patients are actors trained to give specific responses to a certain medical condition that can be reliably replicated between learners. Computer simulation is an interactive program that allows the learner to practice patient care and receive feedback on their medical management. Part-task simulation is a device used to teach a specific skill or procedure such as the placement of a chest tube or delivery of a baby. The high-fidelity mannequin simulator (HFMS) is a dynamic, computer-controlled, full-sized, simulated mannequin capable of giving a history, recreating physical exam findings such as normal and abnormal heart sounds, lung sounds, and pupil findings, as well as physiologic changes including blood pressure, heart rate, and breathing. Some HFMS are even capable of physiologically responding to medication and oxygen administration, receiving electrical cardioversion and procedures such as diagnostic peritoneal lavage and central lines.

## TYPES OF SIMULATORS

### Cardiopulmonary resuscitation simulator

The Resusci-Annie™, developed in 1960, was the earliest form of medical simulation.[15] This part-task simulator was developed from the need for a realistic model for training in basic cardiopulmonary resuscitation (CPR). This is also called “One P” simulator. In a series of studies performed on curarized volunteers, Elam and Safar demonstrated that mouth-to-mouth ventilation could provide adequate oxygenation and elimination of carbon dioxide. Safar later met a toymaker named Asmund Laerdal, who was renowned for his lifelike toy dolls, and commissioned him to create the first CPR manikin, the Resusci-Annie™. Currently, the Resusci-Annie™ is used for basic life support and ACLS training internationally. While this is a powerful tool for basic life support training, it lacks the haptic feedback plus diagnostic and treatment cues of modern simulators.

### Harvey cardiology simulator

In 1976, the first prototype was completed and named after Dr. W. Proctor Harvey, a Professor from Georgetown University, for his innovations in education and teaching. The “Harvey” CPS is a life-size manikin torso capable of reproducing bedside physical findings of a multitude of cardiovascular diseases. In addition, it lacks the ability to simulate therapeutic interventions and physician-patient interactions. Regardless, it is an excellent assessment tool and provides a valuable adjunct to medical education.

### Procedural simulators

Simulation can also be used for training in emergency cardiac procedures. Although several mannequin-based simulators on the market today are equipped with the capacity to perform pericardiocentesis, use of temporary and transvenous pacemakers.

### High-fidelity mannequin simulators

Mannequin simulators provide some of the most realistic and high-yield environments for trainees. These simulators are computer-operated patients capable of recreating almost any disease entity. The first mannequin simulator was developed by Abrahamson and Denson in the early 1960s and was initially used for anesthesia training.[16] Today, these simulators are used in multiple other disciplines. Unlike the part-task simulators mentioned above, the mannequin simulator creates a comprehensive environment for trainees. Multiple studies have shown this method of training to be perceived as realistic and useful.[17–20] these simulators are physiologically modeled to respond appropriately to medications and inhaled gases. Participants speak to the patient, take a complete history and physical exam, and work in a team as they would in a real clinical encounter. Hi-fidelity simulation is rapidly becoming accepted as an advanced teaching tool for medical providers in all stages of practice. Simulation can be used to demonstrate physical findings that would not be possible by other means.[21,22] Traditionally, trained patient actors are used earlier in skill labs to evaluate medical students’ ability to perform a physical exam and assess their ability to interact with patients. Simulation was used during a year-end “high stakes” clinical performance exam (CPX). Simulation provides superior accuracy in determining student diagnostic capabilities than traditional card-based physical finding descriptions.[23–25] On surveying, Peckler *et al*. revealed that the students did not feel comfortable with the simulators and were distracted by them. They remarked the simulators offered less realism and that it was difficult to communicate with them. Not surprisingly, few students wanted future simulation examination, but 38% were open to future simulation-based exams. The result were students like using the simulator for learning, but testing may be problematic.[26,27] While some of the benefits of using this method of training are apparent, the literature has outlined some specific advantages in the education of cardiac emergencies. Wayne and colleagues performed a retrospective evaluation of second-year internal medicine residents who received a simulation-based educational intervention with routine clinical education for cardiac arrest teams.[28] They developed checklists for six common American Heart Association ACLS scenarios and abstracted adherence to these scenarios from the medical record. Simulator-trained residents were seven times more likely to adhere to the ACLS scenarios than non-simulator-trained residents. This study concluded that simulator-based education improved the quality of care delivered by cardiac arrest teams.

Another study reported an improvement in ACLS skills of residents following a simulator-based program.[29] this was a randomized control study that evaluated residents based on their adherence to ACLS protocols. Participants were randomized to either a traditional- or simulator-trained arm. They were evaluated at 3 and 6 months following training. Again, this study found an improvement in residents who participated in simulations. These improvements were sustained with little decay over the study period. In a separate study, no decay was seen in ACLS skills over a 14-month period following simulation-based training.[30] Based on this work; it appears that simulation enhances performance to a greater degree than clinical experience alone.

Mannequin simulators are generally accepted as valuable training tools. Equally important to training in education is the need to evaluate trainees. Mannequin simulators are being used increasingly in this capacity.[30–33] Simulated cases can be tailored to assess the individual Accreditation Council for Graduate Medical Education core competencies.^27^ Some debate exists over which assessment methods are best. To date, there are no validated evaluation tools. At least one study suggests the objective structured clinical examination as a feasible method for trainee assessment.[33] this method showed comparable scores on simulator exams versus oral exam controls at various levels of training. HFMS are also useful for team training. In 1999, the Institute of Medicine published the book “To Err Is Human”, which urged the establishment of multidisciplinary team training. Shapiro and colleagues conducted a study using emergency medicine attending, residents, and nurses to evaluate the use of a simulator to improve team training. Although no statistical differences between experimental and control groups were found, the experimental group showed a trend toward improved team behavior.[35]

## NEED AND DEVELOPMENT OF NEW TRAINING METHODS

### Human patient simulators

The very recent advent of human patient simulators (HPS) promises to improve drastically the intensity and quality of medical training at all levels of proficiency.[36,37] HPS are computer-operated, life-size mannequins capable of physiologically faithful reproduction of human disease signs typically encountered as a part of a medical emergency. The outputs of the device provide realistic chest and heart sounds, pulses, pupillary, and laryngeal reflexes, and allow monitoring all vital signs in a manner identical to the clinical setting. Fully equipped HPS permits execution of several procedures, e.g., intubation, insertion of drainage tubes and catheters, relief of pneumothorax, cricothyroidotomy. Successful implementation of appropriate procedure is immediately reflected in appropriate physiological response (e.g., relief of pneumothorax restores chest sounds and chest excursion on the affected side, normalizes ECG and blood gas status, etc.). Moreover, virtually all drugs used at the prehospital and ER/OR levels can be administered either in the form of intravenous drips or as syringe-injected bolus. Drug treatment of HPS causes correct, dose-dependent systemic response. Importantly, improper or delayed implementation of the required intervention may result in a “fatality.” Hence, the student is simultaneously exposed to the realism of the event (severely ill patient), the demand for instantaneous marshaling of all intellectual resources required to perform the initial diagnosis, and to the demand o execute correct intervention. Yet, just as a paramedic or a physician either in the field or at the emergency room, the student may also feel the ever-present stress of the time limit. The sophisticated nature of HPS devices and their capability of reproducing acute disease in a highly realistic manner that offers virtually life-like challenges led to proposals to use them both in routine training and as tools for competency testing.[31–33] Largely preliminary and, with few exceptions, primarily qualitative reports indicate the beneficial nature of training using HPS.[41,42] Importantly, in addition to individual training, medical team training and preparation can be also executed using these devices.[43,44] Simulation has been also used to study the sources of inaccuracies in reporting critical anesthetic incidents.[44]

The importance of simulation in biomedical education and training increases very rapidly as a substitute for “on-the-job” training and its inadequacies.[45] It is not surprising that the number of HPS continuously increases with over 100 units in the USA alone (nearly 170 worldwide.) However, the cost of HPS may be prohibitive (depending on the sophistication of the model) and at the present only the most affluent institutions can afford them. Moreover, the majorities of the available devices are stationary and, due to their delicate nature, require considerable maintenance by skilled technical personnel.[40]

The very recent HPS models developed by METI and Laerdal are fully mobile. Hence, the machines are unavailable to the majority of those who may need simulator most, i.e. the medical personnel (EMTs, nurses, and physicians) operating in the resource austere environments, and who have the greatest practical difficulties in reaching advanced training centers in order to refresh their professional knowledge and skills. In practice, the very high cost of acquiring multiple HPS units to be placed in such locations is prohibitive. Thus, in order to make human patient simulation accessible to a larger number of the trainees, a completely new approach to HPS-based training has been developed which incorporates extensive use of the Internet-based telepresence and remote access to and the control of the simulating unit.

## RECENT REVIEW OF THE LITERATURE ABOUT SIMULATION USE IN RESUSCITATION TRAINING

In their pilot study Marisa B. Brett-Fleegler assess pediatric residents’ resuscitation competency and evaluate the tool’s reliability, and preliminarily using computerized simulation demonstrated good inter-rater reliability within each domain and for summary scores. Performance analysis shows trends toward improvement with increasing years of training, providing preliminary construct validity.[45] A prospective, observational study was conducted of 34 consecutive hospital-based mock codes by simulation of in-hospital pediatric medical emergencies and cardiopulmonary arrests. A mannequin or computerized simulator was used to enact unannounced, simulated crisis situations involving children with respiratory distress or insufficiency, respiratory arrest, hemodynamic instability, and/or cardiopulmonary arrest. It was found that simulation of pediatric crises can identify targets for educational intervention to improve pediatric cardiopulmonary resuscitation and, ideally, outcomes.[46] The novel teaching approach involved having students actively participate in an unsuccessful resuscitation of a high fidelity human patient simulator with a gunshot wound to the chest. This novel approach was well received and resulted in improvement over baseline.[47] Another paper describes the planning and evaluation of a multi-professional, full-scale simulation-based course for hospital professionals. The use of a systematic approach was successful in the development of this multi-professional full-scale simulation-based educational program, which has proven to be easily applicable and usable.[48]

In a study, the use of a hi-fi simulation mannequin was compared with a standard plastic mannequin when teaching the megacode portion of the neonatal recitation program (NRP) on hi-fi mannequin (SimBaby, Laerdal Medical Corporation, USA) or a traditional plastic mannequin (ALS Baby, Laerdal Medical Corporation, USA). Residents randomly assigned to the hi-fi mannequin did not have improved written scores or improved intubation times. This pilot study demonstrated that a hi-fi mannequin can be used as part of an educational program, such as the NRP. The use of this technology in neonatal resuscitation training is well received by learners and may provide a more realistic model for training.[49]

On assessing the role of ward-based simulation in cardiopulmonary resuscitation training, many of the positive claims made about the value of simulation in such contexts were found. Further analysis indicated that, as a learning resource, simulation compares well with real-life experience, but is most effective when deployed alongside frequent updates based on more traditional teaching methods.[50] Researcher also identify the most common performance deficiencies in paramedics’ management of three simulated pediatric emergencies. Scenarios included an infant cardiopulmonary arrest, sepsis/seizure, and child asthma/respiratory arrest: multiple deficiencies in paramedics’ performance of pediatric resuscitation skills were objectively identified using three manikin-based simulations.[51]

To know regarding importance of team building during CPR studied by dividing rescuer randomized into two different versions of a simulated witnessed cardiac arrest, it was found that team-building has thus to be regarded as an additional task imposed on teams forming adhoc during CPR.[52] Crisis resource management (CRM) skills are a set of nonmedical skills required to manage medical emergencies. There is currently no gold standard for evaluation of CRM performance. Construct validity seems to be present when using both the Ottawa GRS and CRM checklist to evaluate CRM performance during simulated emergencies. Data also indicate the presence of moderate inter-rater reliability when using both the Ottawa GRS and CRM checklist.[53] Objective of this study was to test acceptance of, and interest in, a newly developed prototype of virtual reality enhanced mannequin (VREM), Laerdal Heart Sim 4000 mannequin. Overall, the evaluation of the system was very positive, as was the feeling of immersion and realism of the environment and simulation. Overall, 84.6% of the participants judged the virtual reality experience as interesting and believed that its development could be very useful for healthcare training. The prototype of the virtual reality-enhanced mannequin was well liked, without inference by interaction devices, and deserves full technological development and validation in emergency medical training.[54] Simulation has taken many forms in medicine including, so-called high fidelity simulations resembling as much as possible the actual clinical situations. These forms of simulation have been used to teach the important skill of clinical decision making as well as technical procedures.[55]

Simulating real-world medical emergencies can help medical staff prepare for actual events. When a newly constructed hospital employed simulation to familiarize its cardiac resuscitation team (Code Blue Team) with the layout of the facility, researchers were able to identify several measures that could improve staff response times and patient safety. The authors suggest that a classroom-based orientation for a new facility is insufficient for ensuring that staff can rapidly reach a location where a code was being called. The simulation also uncovered the problems of nonfunctioning overhead speakers, locked stairways, and elevators that did not let passengers override other floor requests that prevented Code Blue Team members from promptly responding to a code. Practice guidelines indicate that the shorter the time a patient must wait to be resuscitated, the better his or her chance of survival.[56] Simulation training has been shown to improve the performance of cricoid pressure on a simulator, but whether simulation training improves the clinical performance of cricoid pressure was unknown. Simulation training with force feedback significantly improved the performance of cricoid pressure in the clinical setting. Simulation training should be used more frequently to train and maintain resuscitation skills, and to compare two different ways of learning (self-study vs. simulation sessions) the adequate steps to resuscitate a neonate in the 5^th^ year undergraduate medical curriculum.[57] Simulation-based training of medical students in management of neonatal resuscitation do not led to significant differences on short-term knowledge comparing with a traditional method.[58] A brief overview of procedural simulators and part-task trainers is also presented, contrasting the two domains and suggesting that a thorough history of the 20+ types of simulator technologies would provide a useful overview and perspective. There has been relatively little cross fertilization of ideas and methods between the two simulator domains. Enhanced interaction between investigators and integration of simulation technologies would be beneficial for the dissemination of the concepts and their applications.[59] Using emergency care simulator (ECS) combined with problem-based learning (PBL) in teaching CPR technique directly involves the medical students in “emergency practice,” resolving of “all sorts of problems,” enhancing emergency awareness, and emergency skill. It can improve teaching quality significantly that is in accordance with the development of modern medicine.[60] Reductions in clinical exposure at both undergraduate and postgraduate levels have been implicated in junior doctors’ inability to recognize and manage critically ill patients. Simulation is used as a central training tool in contemporary advanced life support teaching. Simulation provides a learning opportunity for controlled clinical practice without putting patients or others at risk. The role of task trainers, high and low fidelity patient simulators, and computer-assisted simulation as teaching tools are discussed.[61]

Upon debriefing, it was determined that the previous training influenced execution of the following steps: rapid problem recognition, prompt initiation of specific therapy in the setting of supportive advanced cardiac life support measures, and coordinated team efforts. Although the true cause of efficient resuscitation and ultimate recovery cannot be proven, the efficiency of the resuscitation process, including timely administration of lipid emulsion, is evidence that simulation may be useful for training providers to manage rare emergencies. Simulation education is increasingly used to provide realistic training opportunities for management of clinical situations. Through experiential learning, trainees can acquire skills used in the management of events rarely encountered in practice, such as local anesthetic-induced cardiac toxicity. Advocates of simulation education for rare clinical situations refer to its use in personnel training for disaster medicine, chemical warfare casualties, and for infrequently encountered emergencies in neonatal and pediatric anesthesia.[62,63] Evidence that simulation-based education allows trainees to transfer learned skills from rehearsal to reality is extremely difficult to acquire, especially in the case of rare emergencies. At present, the best data demonstrate only that measurement of simulation scenario performance is improved by simulation scenario training, a not very surprising outcome.[64,65] The translation of skills learned during simulation training to real clinical situations has not been well documented.

## CONCLUSION

There are multiple areas where simulation appears to be used to train physicians in emergencies. The obvious use for decades has been to teach the lay public the techniques of basic life support using CPR mannequins. High-fidelity mannequin simulation appears to have the most promise in the training of physicians and may affect true patient outcome. Programs are using HFMS to train many of the skills needed during the resuscitation of the critically ill patient prior to going to the bedside. The standardization of simulation to train and maintain skills used in ACLS may improve care given to patients during times of decreased and less senior staffing. Finally, HFMS can be used to ensure that all physicians training in the care for the acutely ill, such as emergency medicine, have received appropriate exposure and assessment in all relevant cardiac emergencies prior to their graduation to get better outcome of cardiac arrest resuscitation.
